# Efficacy and safety of intermittent theta-burst stimulation versus continuous theta-burst stimulation for major depressive disorder and bipolar depression: a systematic review

**DOI:** 10.3389/fpsyt.2026.1656576

**Published:** 2026-02-04

**Authors:** Zhi Li, Zhan-Ming Shi, Xin-Hu Yang, Zhi-Ang Su, Wei Wei, Zhen-Juan Qin, Xian-Jun Lan, Xin Wei, Wei Zheng

**Affiliations:** 1 The Brain Hospital of Guangxi Zhuang Autonomous Region, Liuzhou, China; 2 Chongqing Jiangbei Mental Health Center, Chongqing, China; 3 The Affiliated Brain Hospital, Guangzhou Medical University, Guangzhou, China; 4 Guangzhou Medical University, Guangdong Engineering Technology Research Center for Translational Medicine of Mental Disorders, Guangzhou, China

**Keywords:** bipolar depression, continuous theta-burst stimulation, intermittent theta-burst stimulation, major depressive disorder, systematic review

## Abstract

**Background:**

The therapeutic efficacy and safety profiles of daily or accelerated intermittent theta-burst stimulation (iTBS) in comparison to daily or accelerated continuous theta-burst stimulation (cTBS) for patients with major depressive disorder (MDD) or bipolar depression (BD) remain inadequately explored, respectively. This systematic review evaluates the efficacy, safety, and tolerability of daily or accelerated iTBS compared to daily or accelerated cTBS in patients with MDD or BD.

**Methods:**

A comprehensive search was conducted in both Chinese (WanFang, China National Knowledge Infrastructure) and English (PubMed, EMBASE, PsycINFO, Cochrane Library) databases to identify randomized controlled trials (RCTs) examining the efficacy and safety of daily or accelerated iTBS compared to daily or accelerated cTBS in patients with MDD or BD.

**Results:**

Three studies (n = 87) investigated the efficacy, safety, and tolerability of daily iTBS versus daily cTBS (1 RCT, n = 30) in patients with MDD, and accelerated iTBS versus accelerated cTBS (2 RCTs, n = 57) in patients with MDD or BD. In patients with MDD, daily iTBS outperformed daily cTBS in terms of antidepressant efficacy, with comparable safety and tolerability profiles (1 RCT). Accelerated cTBS demonstrated superior anxiolytic (1 RCT) and anti-suicidal efficacy (1 RCT) compared to accelerated iTBS in MDD, with similar antidepressant efficacy, safety, and tolerability profiles for patients with MDD or BD (2 RCTs).

**Conclusion:**

Among the limited number of available studies, the efficacy and safety of daily or accelerated iTBS compared to daily or accelerated cTBS in patients with MDD or BD were uncertain. Future research with larger sample sizes and standardized assessments is essential to confirm these findings.

## 1 Introduction

Major depressive disorder (MDD) and bipolar depression (BD) are severe mental health conditions characterized by persistent sadness, anhedonia, and significant impairment in neurocognitive and daily functioning (1, 2). Due to their chronic nature, these disorders impose a substantial burden on patients, caregivers, and society (3, 4). The prolonged course of MDD and BD contributes to a diminished quality of life, an increased risk of comorbidities, and greater economic strain (5, 6). Effective treatment of MDD and BD could enhance interpersonal relationships, reduce relapse risk, and facilitate social functioning (7–10).

Psychopharmacological interventions have been the cornerstone of MDD and BD treatment (11, 12). However, antidepressants exhibit an efficacy rate of only 54% (13), with at least 30% of patients with MDD or BD developing treatment-resistant depression (TRD) (14). Although novel antidepressants, including psilocybin (15) and ketamine (16), have shown potent antidepressant effects, their long-term safety profiles remain uncertain. Given the risks and the urgent need for more effective treatments for MDD and BD, the development of neurostimulation interventions is critical.

Neurostimulation interventions such as repetitive transcranial magnetic stimulation (rTMS) have been investigated as standalone treatments as well as augmentation strategy for MDD and BD in clinical practice (17). However, conventional rTMS requires 37.5 minutes per session and multiple weeks of treatment to achieve a clinical response in these conditions (17). Accelerated TMS, defined as a protocol delivering more than one daily TMS session, is one emerging delivery schedule of TMS aimed to reduce treatment duration and improve response time, with the goal of achieving similar (or superior) levels of efficacy (18). To alleviate the time burden of rTMS, new protocols with higher dosing frequencies and multiple daily sessions, such as theta-burst stimulation (TBS), have been developed (19, 20). TBS involves delivering three magnetic pulses at a 20-ms interval (50 Hz), repeated every 200 ms to align with the 5-Hz theta rhythm, inducing more robust and lasting alterations in cortical excitability (21). Intermittent TBS (iTBS) induces long-term potentiation (LTP)-like effects, whereas continuous TBS (cTBS) results in long-term depression (LTD)-like reductions in cortical excitability (22). Daily or accelerated (≥2 sessions/day) TBS (e.g., iTBS or cTBS) effectively modulates brain activity and influences mood circuits, offering a powerful treatment alternative for patients with MDD or BD (23, 24).

The effectiveness, safety, and tolerability of daily or accelerated iTBS compared to daily or accelerated cTBS for MDD and BD remain uncertain. Several studies comparing these two protocols had yielded mixed findings (25–27). For instance, Li et al. demonstrated that daily iTBS was more effective in alleviating depressive symptoms than daily cTBS in patients with MDD (26), while other studies indicated that the antidepressant effects of accelerated iTBS were comparable to those of accelerated cTBS in patients with MDD or BD (25, 27). Several systematic reviews and meta-analyses (24, 28, 29) have assessed the efficacy, safety, and tolerability of TBS for patients with MDD or BD, concluding that TBS was more effective than standard rTMS (28) and sham stimulation (24, 29). A recent network meta-analysis (24) showed a significant superiority of active over sham iTBS in improving depressive symptoms in depression, while cTBS alone had no therapeutic efficacy in depression. However, this meta-analysis (24) did not include a recent randomized controlled trial (RCT) (27) that directly compared the two protocols in MDD. To fill this gap, a systematic review of head-to-head RCTs examining the efficacy and safety of daily or accelerated iTBS compared to daily or accelerated cTBS for patients with MDD or BD was conducted. This systematic review seeks to offer a more comprehensive assessment of the comparative effectiveness and safety of these two stimulation methods using daily or accelerated protocols, respectively.

## 2 Material and methods

### 2.1 Search strategy and selection criteria

Three researchers (ZL, ZMS, and ZAS) independently performed a thorough literature search across four international databases (PubMed, EMBASE, PsycINFO, and Cochrane Library) and two Chinese databases (WanFang database and China National Knowledge Infrastructure), covering their inception to February 16, 2025. For example, the search strategy for PubMed was as follows: (“transcranial magnetic stimulation”[Mesh] OR transcranial magnetic stimulation OR trans-cranial magnetic stimulation OR rTMS OR TMS OR theta-burst stimulation OR theta burst transcranial magnetic stimulation OR transcranial theta burst stimulation OR TBS OR intermittent theta-burst stimulation OR intermittent theta burst stimulation OR (intermittent* AND theta burst stimulation) OR iTBS) OR (continuous theta-burst stimulation OR continuous theta burst stimulation OR (continuous* AND theta burst stimulation) OR cTBS) AND (“depression”[Mesh] OR “depressive disorder”[Mesh] OR depressive disorder* OR depressive neuros* OR endogenous depression* OR unipolar depression* OR depressive syndrome* OR neurotic depression* OR depress* OR dysphor* OR melanchol* OR antidepress*). Additionally, reference lists of eligible studies (25–27) and related systematic reviews or meta-analyses (24, 28–30) were examined for potential inclusion.

In accordance with the Preferred Reporting Items for Systematic Reviews and Meta-Analyses (PRISMA) guidelines (31), study inclusion criteria were established based on the *PICOS* framework. *P*articipants: adult patients diagnosed with MDD or BD, including those with TRD, according to any internationally recognized diagnostic criteria. For example, TRD is defined by failure to respond to at least two prior adequate antidepressant trials (26). *I*ntervention *vs. C*omparison: 1) daily iTBS plus treatment as usual (TAU) (e.g., stable medication regimens) versus daily cTBS plus TAU; and 2) accelerated iTBS plus TAU versus accelerated cTBS plus TAU. *O*utcomes: the primary outcome was the change in depressive symptoms, evaluated using standardized depression scales, such as the Hamilton Depression Rating Scale (HAMD) (32). Secondary outcomes included changes in anxiety symptoms, suicidal ideation, neurocognitive function, discontinuation rates, and adverse events (e.g., dizziness and headache). Study design: only published RCTs assessing the efficacy, safety, and tolerability of daily or accelerated iTBS compared to daily or accelerated cTBS in patients with MDD or BD were considered. Studies with randomized within-subjects designs (33, 34), retrospective studies, and case reports/series were excluded. For multiple publications based on the same dataset (26, 35, 36), only the study with the most comprehensive data was included (26).

### 2.2 Data extraction

Data extraction was independently conducted by the same three researchers (ZL, ZMS, and ZAS) using a standardized form, which captured study authorship, publication year, design, TBS protocol, and both primary and secondary outcomes. When additional information was required, corresponding authors were contacted *via* email. Disagreements were resolved through consensus among the researchers (ZL, ZMS, and ZAS), with the senior researcher (WZ) consulted when necessary.

### 2.3 Study quality assessment

The quality of each included RCT was independently evaluated by the same three investigators (ZL, ZMS, and ZAS) using the Jadad scale (37) and the Cochrane Risk of Bias tool (38). Studies scoring ≥3 on the Jadad scale were classified as high-quality (39).

## 3 Results

### 3.1 Literature search

**Figure 1:**
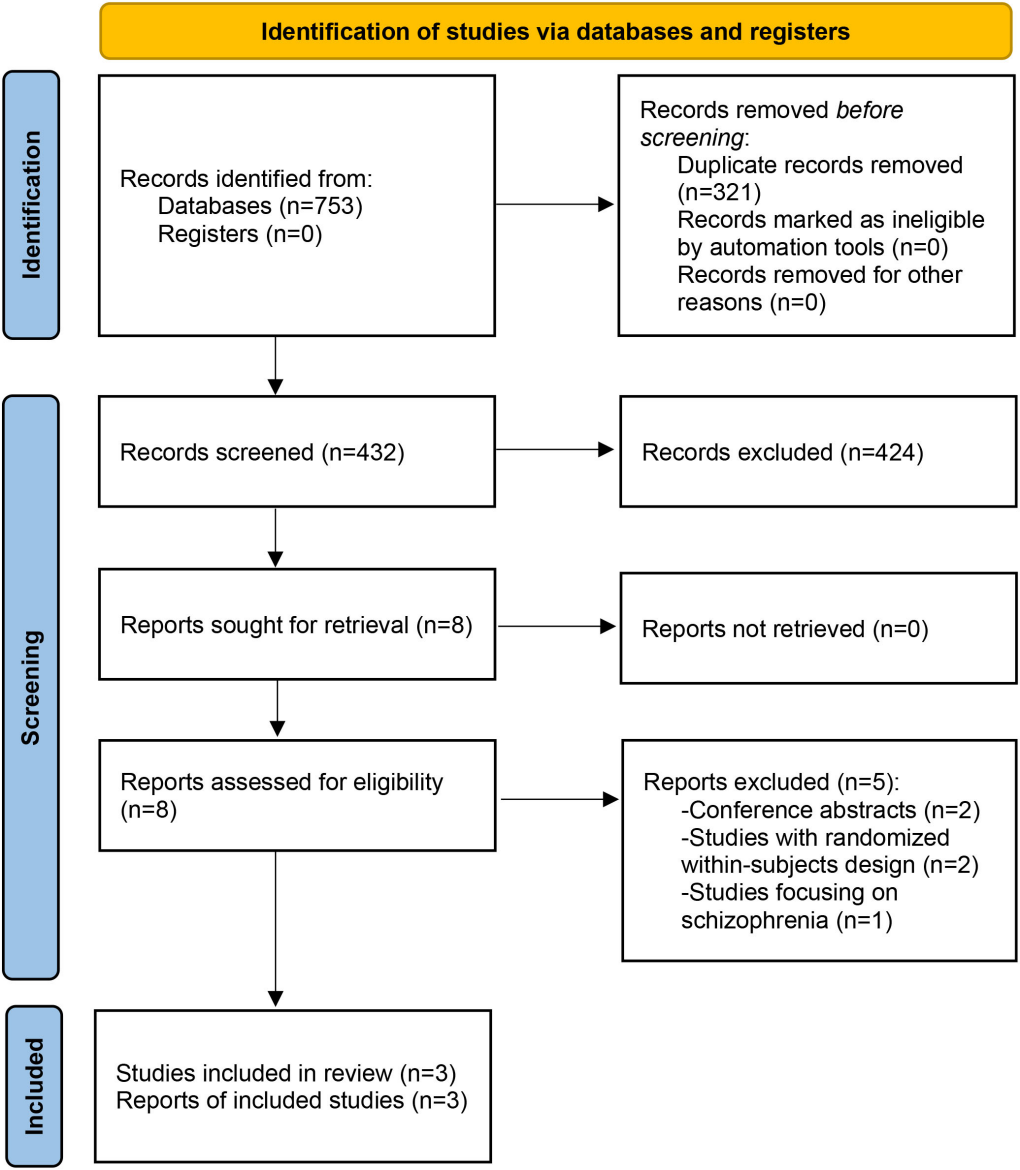
PRISMA flow diagram. PRISMA, Preferred Reporting Items for Systematic reviews and Meta-Analyses.

As shown in **Figure 1**, a total of 432 studies were identified. After screening titles, abstracts, and full texts, three RCTs (25–27) met the inclusion criteria. Due to insufficient data, a meta-analysis could not be conducted.

### 3.2 Study characteristics

**Table 1** summarizes patient characteristics and TBS protocols for each included RCT. These three RCTs, published between 2010 and 2024, involved 87 participants. One RCT (n = 30) compared daily iTBS to daily cTBS in patients with MDD, while two RCTs (n = 57) assessed accelerated iTBS versus accelerated cTBS in patients with MDD or BD. The weighted mean age of participants was 34.1 years, with males representing 28.7% of the sample (range: 15.4%–40.0%). Daily or accelerated iTBS was applied to the left dorsolateral prefrontal cortex (L-DLPFC), and daily or accelerated cTBS was applied to the right DLPFC (R-DLPFC). The intensity for daily iTBS/cTBS was set at 80% motor threshold (MT), delivering 18,000 pulses (26). For accelerated iTBS or cTBS, the stimulation intensity ranged from 90% to 100% MT, with a pulse range of 12,000–90,000 (25, 27). Treatment durations ranged from five days to two weeks (**Table 1**).

**Table 1:** Patient characteristics and iTBS or cTBS parameters of each included RCT.

Studies (country)	N^a^	-Diagnostic criteria -Setting (%) -Diagnosis (TRD %)	-Illness duration: years^b^ -Male^a^ (%) -Age: years, (range)	-iTBS groups (number of participants) -cTBS groups (number of participants)	-Treatment duration -Treatment site	-Intensity (% MT) -Frequency (Hz) -Coil type		-Train duration (s) -Intertrain interval (s)		-Pulses per session -Number of sessions, sessions daily (n) -Total pulses		Jaded score
						iTBS	cTBS	iTBS	cTBS	iTBS	cTBS	
Daily iTBS versus daily cTBS (1 RCT, n = 30)												
Li et al., 2014 (China) (26)	30^c^	**-**DSM-IV **-**NR **-**MDD (100)	**-**NR **-**12 (40.0) **-**45.8 (25-64)	-Daily iTBS + TAU^d^ (15) -Daily cTBS + TAU^e^ (15)	-2 weeks -iTBS: L-DLPFC cTBS: R-DLPFC	**-**80% AMT **-**50 Hz triplets at 5 Hz **-**F8-coil	**-**80% AMT **-**50 Hz triplets at 5 Hz **-**F8-coil	**-**2 **-**8	**-**120 **-**0	**-**1800 **-**10 (1) **-**18000	**-**1800 **-**10 (1) **-**18000	4
Accelerated iTBS versus accelerated cTBS (2 RCTs, n = 57)												
Chistyakov et al., 2010 (Israel) (25)	13^f^	**-**DSM-IV **-**Inpatient (100) **-**MDD (NR) + BD (NR)	**-**21.0 **-**2 (15.4) **-**54.7 (20-75)	-Accelerated iTBS + TAU^g^ (7) -Accelerated cTBS + TAU^g^ (6)	-10 days -iTBS: L-DLPFC cTBS: R-DLPFC	**-**90% AMT **-**50 Hz triplets at 5 Hz **-**F8-coil	**-**90% AMT **-**50 Hz triplets at 5 Hz **-**F8-coil	**-**2 **-**8	**-**40 **-**0	**-**600 **-**20 (2) **-**12000	**-**600 **-**20 (2) **-**12000	2
Zhao et al., 2024 (China) (27)	44	**-**DSM-5 **-**Inpatient (88.6) and outpatients (11.4) **-**MDD (100)	**-**2.6 **-**11 (25.0) **-**20.1 (13-40)	-Accelerated iTBS + TAU^h^ (22) -Accelerated cTBS + TAU^h^ (22)	-5 days -iTBS: L-DLPFC cTBS: R-DLPFC"	**-**100% RMT **-**50 Hz triplets at 5 Hz **-**F8-coil	**-**100% RMT **-**50 Hz triplets at 5 Hz **-**F8-coil	**-**2 **-**8	**-**120 **-**0	**-**1800 **-**50 (10) **-**90000	**-**1800 **-**50 (10) **-**90000	3

a Data were extracted based on random assignment.

b Available data were reported as means.

c Of the total sample of 60 participants, 30 were randomized to receive either iTBS or cTBS.

d Patients were required to maintain their original medication regimens, except for four patients who were drug-free.

e Patients were required to maintain their original medication regimens, except for five patients who were drug-free.

f Of the total sample of 33 participants, 13 were randomized to receive either iTBS or cTBS.

g Patients were maintained on their previous medications throughout the course of TBS treatment.

h Patients were required to remain on a stable antidepressant regimen throughout the study.

AMT, active motor threshold; BD, bipolar depression; cTBS, continuous theta-burst stimulation; DSM-IV, Diagnostic and Statistical Manual of Mental Disorders 4^th^ edition; DSM-5, Diagnostic and Statistical Manual of Mental Disorders 5^th^ edition; F8, figure-of-eight; iTBS, intermittent theta-burst stimulation; L-DLPFC, left dorsolateral prefrontal cortex; MDD, major depressive disorder; MT, motor threshold; N, number of patients; NR, not reported; RCT, randomized controlled trial; R-DLPFC, right dorsolateral prefrontal cortex; RMT, resting motor threshold; TAU, treatment as usual; TRD, treatment-resistant depression.

### 3.3 Quality assessment

**Figure 2:**
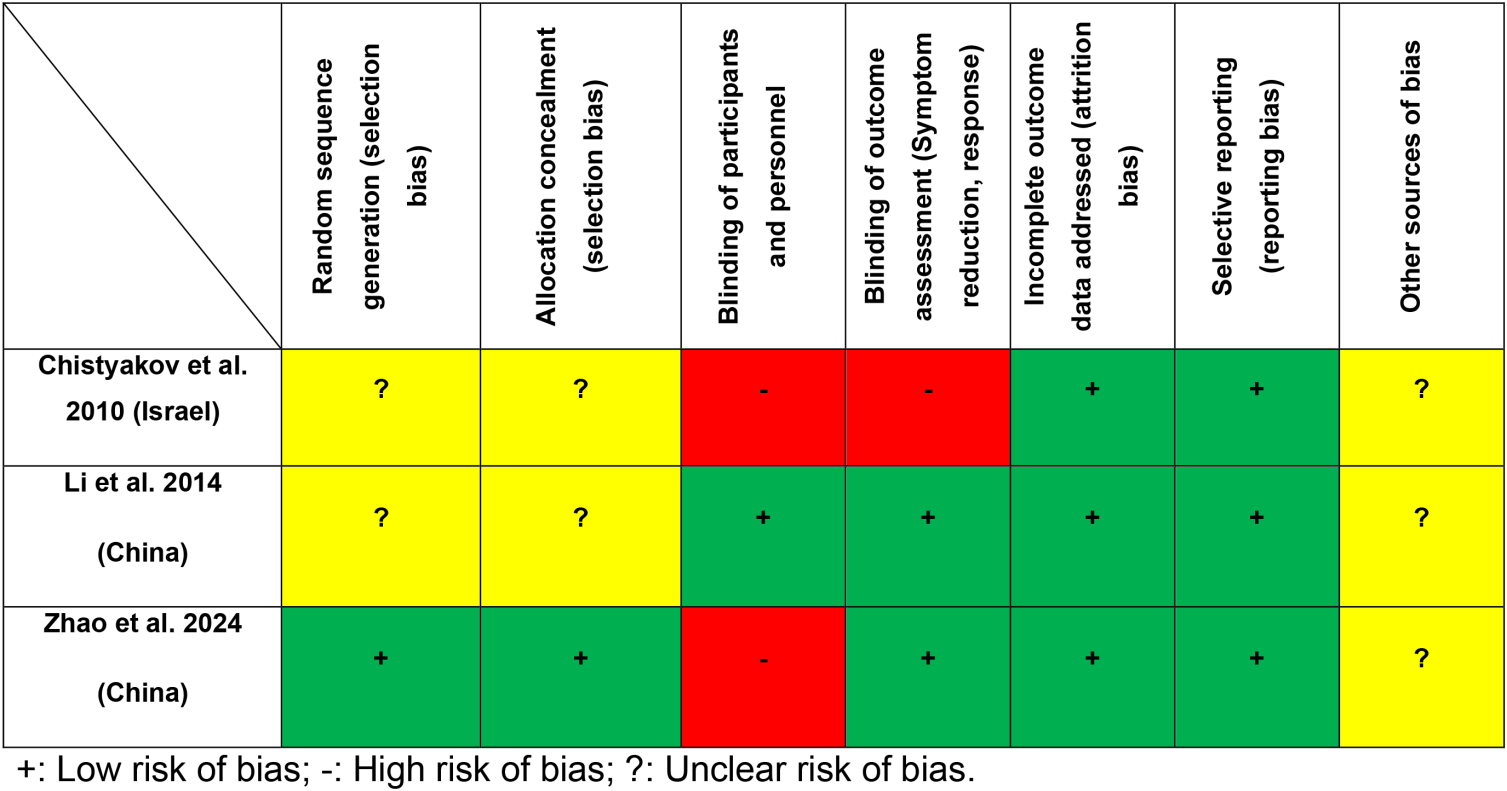
Cochrane risk of bias.

Quality assessment using the Cochrane Risk of Bias tool revealed that all RCTs (3/3, 100%) had a low risk of bias for incomplete outcome data and selective reporting. Two studies (2/3, 66.7%) showed a low risk of bias for blinding of outcome assessment, while one RCT (1/3, 33.3%) demonstrated a low risk of bias for random sequence generation, allocation concealment, and blinding of participants and personnel (**Figure 2**). The weighted mean Jadad score was 3.0 (range: 2–4), with two RCTs (66.7%) classified as high quality (Jadad score ≥3) (**Table 1**).

### 3.4 Changes in clinical symptoms

#### 3.4.1 Daily iTBS versus daily cTBS

A single RCT (26) evaluated the antidepressant efficacy of daily iTBS versus daily cTBS in patients with MDD, demonstrating a significant superiority of daily iTBS over cTBS in alleviating depressive symptoms (**Table 2**).

**Table 2:** iTBS versus cTBS for patients with MDD or BD: clinical efficacy.

Intervention studies	Assessment scales	Findings
Daily iTBS versus daily cTBS (1 RCT, n = 30)		
Li et al., 2014 (China) (26)	HAMD-17	A significant superiority of daily iTBS over daily cTBS was observed in improving depressive symptoms, as measured by the HAMD-17, in patients with TRD.
Accelerated iTBS versus accelerated cTBS (2 RCTs, n = 57)		
Chistyakov et al., 2010 (Israel) (25)	HAMD	No significant group difference was observed in the improvement of depressive symptoms, as measured by the HAMD, in patients with MDD or BD.
Zhao et al., 2024 (China) (27)	HAMD-24, HAMA, BSI	While accelerated cTBS showed no advantage over accelerated iTBS in improving depressive symptoms, it was significantly more effective in reducing both anxiety symptoms and suicidal ideation in patients with TRD.

BD, bipolar depression; BSI, Beck Scale for Suicide Ideation; cTBS, continuous theta-burst stimulation; HAMA, Hamilton Anxiety Rating Scale; HAMD-17, 17-item version of the Hamilton Depression Rating Scale; HAMD-24, 24-item version of the Hamilton Depression Rating Scale; iTBS, intermittent theta-burst stimulation; MDD, major depressive disorder; RCT, randomized controlled trial; TRD, treatment-resistant depression.

#### 3.4.2 Accelerated iTBS versus accelerated cTBS

Two RCTs (25, 27) (2/2, 100.0%) compared the antidepressant effects of accelerated iTBS and accelerated cTBS in patients with MDD or BD, reporting no significant differences between the groups (**Table 2**). In addition Zhao et al. (27) assessed the anxiolytic and anti-suicidal effects of accelerated iTBS versus accelerated cTBS in patients with MDD. Specifically, their results showed that accelerated cTBS was superior to accelerated iTBS in reducing both anxiety symptoms and suicidal ideation (**Table 2**).

### 3.5 Neurocognitive function

#### 3.5.1 Daily iTBS versus daily cTBS

A single RCT (26) investigated the effects of daily iTBS and daily cTBS on executive function, without direct group comparison (**Table 3**).

**Table 3:** iTBS versus cTBS for patients with MDD or BD: neurocognitive function.

Intervention studies	Assessment scales	Findings
Daily iTBS versus daily cTBS (1 RCT, n = 30)		
Li et al., 2014 (China) (26)^a^	WCST	Daily iTBS and daily cTBS were not directly compared for their neurocognitive effects; however, the findings of this study indicated that daily iTBS, rather than daily cTBS, improved executive function independently of antidepressant effects in patients with TRD.
Accelerated iTBS versus accelerated cTBS (2 RCTs, n = 57)		
Chistyakov et al., 2010 (Israel) (25)	NR	NR
Zhao et al., 2024 (China) (27)	THINC-it	No significant group difference was observed in neurocognitive improvement between accelerated iTBS and accelerated cTBS in patients with TRD.

a The data originate from another article based on the same experiment (see reference 36).

BD, bipolar depression; cTBS, continuous theta-burst stimulation; iTBS, intermittent theta-burst stimulation; MDD, major depressive disorder; NR, not reported; RCT, randomized controlled trial; THINC‐it, THINC-integrated tool; TRD, treatment-resistant depression; WCST, Wisconsin Card Sorting Test.

#### 3.5.2 Accelerated iTBS versus accelerated cTBS

A single RCT (27) examined neurocognitive function between the two groups and found no significant differences (**Table 3**).

### 3.6 Rates of discontinuation and adverse events

#### 3.6.1 Daily iTBS versus daily cTBS

No significant differences in adverse effects (e.g., headache, dizziness, nausea) or discontinuation rates were reported between the groups in Li et al.’s study (all Ps > 0.05) (26) (**Supplementary Table 1**).

#### 3.6.2 Accelerated iTBS versus accelerated cTBS

No significant differences in adverse effects (e.g., discomfort at treatment site, fatigue, headache, dizziness, nausea) or discontinuation rates were observed between the groups in the two included RCTs (all Ps > 0.05) (25, 27) (**Supplementary Table 1**).

## 4 Discussion

This is the first systematic review of three RCTs (25–27) (n = 87) directly comparing the efficacy, acceptability, and safety of daily or accelerated iTBS compared to daily or accelerated cTBS in patients with MDD or BD. The key findings are as follows: 1) insufficient evidence exists to determine the effectiveness of daily iTBS compared to cTBS, as well as accelerated iTBS versus cTBS in alleviating suicidal ideation, anxiety, depressive symptoms, or neurocognitive deficits in patients with MDD or BD; 2) the rates of discontinuation and adverse effects were similar between daily iTBS and daily cTBS (1 RCT); they were also similar between accelerated iTBS and accelerated cTBS (2 RCTs) in patients with MDD or BD.

The efficacy of daily or accelerated iTBS compared to daily or accelerated cTBS for reducing depressive symptoms in patients with MDD or BD was uncertain. Several factors may influence the antidepressant effects of daily or accelerated TBS in patients with MDD or BD. For example, the precision of the stimulation site seems to be a critical consideration (40, 41), and functional targeting might further augment treatment outcomes (27, 41). Generally, iTBS targets the L-DLPFC, which is closely associated with negative emotional judgment, while cTBS targets the R-DLPFC, which is linked to attentional modulation (42). The antidepressant effect of daily iTBS may be attributed to the induction of lasting central effects through the LTP-like effects on neuronal synapses in Li et al.’s study (26). However, under intensive treatment protocols, both modalities may yield comparable outcomes (27). iTBS and cTBS exhibit robust nonlinear plasticity effects (43), and the accelerated protocols may diminish the differential plasticity effects between the two techniques. In Blumberger et al.’s study (44), no significant difference was found between daily iTBS and accelerated iTBS protocols in terms of antidepressant efficacy. Notably, the limited number of RCTs and small sample sizes in this systematic review may partially account for the inconsistent results. Therefore, further well-powered RCTs are necessary to directly compare the efficacy of daily or accelerated iTBS compared to daily or accelerated cTBS in patients with MDD or BD.

In this systematic review, only one RCT assessed the comparative anxiolytic effects of accelerated iTBS versus accelerated cTBS, finding that accelerated cTBS significantly outperformed accelerated iTBS in alleviating anxiety symptoms in patients with MDD (27). Although anxiety and depression are generally considered distinct conditions in diagnostic criteria, anxious depression is a relatively common syndrome (45, 46). However, Zhao et al.’s study did not specifically evaluate the anxiolytic effects of accelerated cTBS over iTBS in patients with anxious depression (27). Anxious depression, compared to non-anxious depression, exhibits distinct neurobiological profiles, including differences in hypothalamic-pituitary-adrenal (HPA) axis function (47), brain structure and function (48, 49), and inflammation markers (50). While anxious depression is associated with poorer treatment outcomes, adjunctive rTMS has shown significant anxiety reduction in such patients while maintaining comparable antidepressant efficacy to non-anxious depression (51). A noninferiority RCT also found comparable anxiolytic effects between iTBS and rTMS (52). Thus, the comparative anxiolytic effects of daily or accelerated iTBS compared to daily or accelerated cTBS in patients with anxious depression warrant further investigation.

Suicide risk is often an exclusion criterion in TBS trials for MDD, leaving suicidal patients with limited treatment options. While accelerated iTBS shows promise in reducing suicidality (53), the evidence base remains inconclusive. In this systematic review, no RCT has assessed the anti-suicidal efficacy of daily iTBS versus daily cTBS, and only one RCT (27) has evaluated the anti-suicidal efficacy of accelerated iTBS versus accelerated cTBS in patients with depression. Consequently, the anti-suicidal effects of both treatment protocols were inadequately established in patients with MDD or BD. Zhao et al.’s study (27) found that accelerated cTBS was significantly more effective than accelerated iTBS in reducing suicidal ideation in MDD patients, as measured by the Beck Scale for Suicidal Ideation. However, scales have limited sensitivity and predictive validity for suicidal behaviors (54, 55), and there is not yet a single instrument considered to be the gold standard for suicide risk assessment (56). When scales such as the Columbia Suicide Severity Rating Scale (57) for suicide risk are used, the results need to be quickly reviewed and followed by a clinical evaluation if the scores suggest a risk (58). The suicide module of the Mini-International Neuropsychiatric Interview was generally agreed upon as a better tool for measuring suicide risk (59, 60). Although accelerated cTBS shows promise in reducing suicidality in patients with MDD (27), further research with larger cohorts and more rigorous methodologies is required to clarify the therapeutic potential of accelerated cTBS in patients with MDD or BD.

Non-invasive brain stimulation (NIBS) techniques may improve certain neurocognitive function in both patients with depression (61, 62) and healthy individuals (63, 64). In this systematic review, two RCTs examined the neurocognitive effects of daily or accelerated iTBS compared to daily or accelerated cTBS in patients with MDD (26, 27). However, the differences in neurocognitive effects of daily or accelerated iTBS compared to daily or accelerated cTBS were uncertain. Li et al. (26) used the Wisconsin Card Sorting Test to compare the neurocognitive effects of daily iTBS and daily cTBS, while Zhao et al. (27) employed the THINC-integrated tool to assess the effects of accelerated iTBS and accelerated cTBS. Notably, Zhao et al.’s study found no significant group differences in neurocognitive function (27). Preliminary evidence suggests that daily iTBS may improve certain neurocognitive functions in patients with MDD (65). A notable increase in left hippocampal grey matter volume in part of the dentate gyrus was observed following accelerated iTBS (66), potentially contributing to enhanced neurocognitive function in patients with MDD. In contrast, cTBS has been shown to impair attention, inhibitory control, planning, and goal-directed behavior, while enhancing decision-making by reducing impulsivity in healthy individuals (67). However, the effects of cTBS on neurocognitive functions in patients with MDD or BD remain uncertain. Although there is currently no gold standard for assessing cognitive impairment in depression (68), neurocognitive assessment tools, such as the MATRICS Consensus Cognitive Battery (69) or the Repeatable Battery for the Assessment of Neuropsychological Status (70), should be employed to evaluate the effects of daily or accelerated iTBS compared to daily or accelerated cTBS on neurocognitive function in patients with MDD or BD.

Both daily and accelerated TBS (i.e., iTBS and cTBS) are considered to be relatively safe as adjunctive therapies for patients with MDD or BD (23, 71) and healthy individuals (72, 73), with no serious adverse events reported. Mild side effects, such as headache, dizziness, nausea, and discomfort, were common. The majority of TBS-related adverse events were mild, affecting 5% of both healthy individuals and clinical patients (74). Given the high-frequency bursts of TBS, there is a potential risk of inducing seizures in patients with MDD or BD. While no seizures were reported in the current systematic review, a case report documented cTBS-induced seizures in a healthy individual (75). Consequently, appropriate preventive measures, including physician supervision and access to emergency medical care, should be in place during daily or accelerated TBS treatments.

Several limitations should be considered when interpreting the findings of this systematic review. First, despite a comprehensive search, only three RCTs with small sample sizes were included. Second, due to significant heterogeneity (such as variations in intervention parameters, patient characteristics (e.g., the use of pharmacologic agents), and treatment duration) among the included studies, a meta-analysis could not be conducted. Therefore, the optimal stimulation protocol of TBS (i.e., iTBS and cTBS) for patients with MDD or BD should be investigated. Third, patients with TRD may respond differently to TBS than patients without TRD, affecting result interpretation. However, only three RCTs were included in this systematic review, making it difficult to compare the efficacy and safety of TBS (e.g., iTBS or cTBS) in patients with TRD versus non-TRD. Fourth, the included RCTs with a varied patient population, such as those with MDD and BD, limited the clarity of these findings. Finally, this systematic review was not registered.

In conclusion, the preliminary evidence available is insufficient to determine the comparative efficacy of daily or accelerated iTBS compared to daily or accelerated cTBS for patients with MDD or BD. Further research with larger sample sizes and standardized protocols is required to validate these findings.

## Data Availability

The original contributions presented in the study are included in the article/**Supplementary Material**, further inquiries can be directed to the corresponding authors.

## Supplementary material

Supplementary Table 1 iTBS versus cTBS for patients with MDD and BD: rates of discontinuation and adverse effects.
