# Supplementary material for: Efficacy and safety of intermittent theta-burst stimulation versus continuous theta-burst stimulation for major depressive disorder and bipolar depression: a systematic review
Source: Front Psychiatry. 2026 Feb 4;17:1656576. doi: 10.3389/fpsyt.2026.1656576 (PMC12914266; doi:10.3389/fpsyt.2026.1656576)
Supplement: Supplementary Table 1 — iTBS versus cTBS for patients with MDD and BD: rates of discontinuation and adverse effects. [file Table1.docx]

**Supplemental Table 1. iTBS versus cTBS for patients with MDD and BD:** **rates of discontinuation and adverse effects.**

| **Intervention Studies** | **Discontinuation rate** | | | | **Adverse events** | | | |
| --- | --- | --- | --- | --- | --- | --- | --- | --- |
|  | **Total (n, %)** | **iTBS (n, %)** | **cTBS (n, %)** | **Significance** |  | **iTBS (n, %)** | **cTBS (n, %)** | **Significance** |
| **Daily iTBS versus daily cTBS (1 RCT, n=30)** | | | | | | | | |
| Li et al., 2014 (China) | 0 (0) | 0 (0) | 0 (0) | NS | Dizziness | 2 (13.3) | 1 (6.7) | NS |
|  |  |  |  |  | Headache | 3 (20.0) | 1 (6.7) | NS |
|  |  |  |  |  | Others (palpitation, nausea) | 2 (13.3) | 0 (0) | NS |
| **Accelerated iTBS versus accelerated cTBS (2 RCTs, n=57)** | | | | | | | | |
| Chistyakov et al., 2010 (Israel) | 0 (0) | 0 (0) | 0 (0) | NS | Slight pain | 2 (28.6) | 0 (0) | NS |
| Zhao et al., 2024 (China) | 4 (9.1) | 2 (9.1) | 2 (9.1) | NS | Discomfort at treatment site | 5 (22.7) | 5 (22.7) | NS |
|  |  |  |  |  | Dizziness | 1 (4.5) | 2 (9.1) | NS |
|  |  |  |  |  | Fatigue | 2 (9.1) | 4 (18.2) | NS |
|  |  |  |  |  | Headache | 3 (13.6) | 2 (9.1) | NS |
|  |  |  |  |  | Nausea | 1 (4.5) | 0 (0) | NS |
| Abbreviations: BD=bipolar depression; cTBS=continuous theta burst stimulation; iTBS=intermittent theta burst stimulation; MDD=major depressive disorder; NS=not significant (*P* > 0.05); RCT=randomized controlled trial. | | | | | | | | |
